# Feeding Honeybee Colonies with Honeybee-Specific Lactic Acid Bacteria (Hbs-LAB) Does Not Affect Colony-Level Hbs-LAB Composition or *Paenibacillus larvae* Spore Levels, Although American Foulbrood Affected Colonies Harbor a More Diverse Hbs-LAB Community

**DOI:** 10.1007/s00248-019-01434-3

**Published:** 2019-09-10

**Authors:** Sepideh Lamei, Jörg G. Stephan, Bo Nilson, Sander Sieuwerts, Kristian Riesbeck, Joachim R. de Miranda, Eva Forsgren

**Affiliations:** 1 Department of Ecology, Swedish University of Agricultural Sciences, Uppsala, Sweden; 2 Clinical Microbiology, Department of Translational Medicine, Lund University, Malmö, Sweden; 3 Swedish Species Information Centre, Swedish University of Agricultural Sciences, Uppsala, Sweden; 4 Clinical Microbiology, Labmedicine, Region Skåne, Lund, Sweden; 5 Department of Laboratory Medicine Lund, Lund University, Lund, Sweden; 6 Arla Innovation Center, Aarhus, Denmark

**Keywords:** American foulbrood, *Apis mellifera*, Beneficial microbes, *Bifidobacterium*, Intestinal microbiota, *Lactobacillus*

## Abstract

The main current methods for controlling American Foulbrood (AFB) in honeybees, caused by the bacterial pathogen *Paenibacillus larvae*, are enforced incineration or prophylactic antibiotic treatment, neither of which is fully satisfactory. This has led to an increased interest in the natural relationships between the pathogenic and mutualistic microorganisms of the honeybee microbiome, in particular, the antagonistic effects of Honeybee-Specific Lactic Acid Bacteria (hbs-LAB) against *P. larvae*. We investigated whether supplemental administration of these bacteria affected *P. larvae* infection at colony level over an entire flowering season. Over the season, the supplements affected neither colony-level hbs-LAB composition nor naturally subclinical or clinical *P. larvae* spore levels. The composition of hbs-LAB in colonies was, however, more diverse in apiaries with a history of clinical AFB, although this was also unrelated to *P. larvae* spore levels. During the experiments, we also showed that qPCR could detect a wider range of hbs-LAB, with higher specificity and sensitivity than mass spectrometry. Honeybee colonies are complex super-organisms where social immune defenses, natural homeostatic mechanisms, and microbiome diversity and function play a major role in disease resistance. This means that observations made at the individual bee level cannot be simply extrapolated to infer similar effects at colony level. Although individual laboratory larval assays have clearly demonstrated the antagonistic effects of hbs-LAB on *P. larvae* infection, the results from the experiments presented here indicate that direct conversion of such practice to colony-level administration of live hbs-LAB is not effective.

**Electronic supplementary material:**

The online version of this article (10.1007/s00248-019-01434-3) contains supplementary material, which is available to authorized users.

## Introduction

*Apis mellifera* is a social insect with a diverse microbiota consisting of dozens of bacterial taxa, including Gram-positive and Gram-negative bacteria as well as alpha-, beta-, and gamma-proteobacteria. Most of these bacteria are not pathogenic, with a few critical exceptions [1–3]. One of these is the spore-forming bacterium *Paenibacillus larvae*, the causative agent of American foulbrood (AFB) which is the most lethal brood disease of honeybees, and ultimately fatal to the colony if left untreated [4]. Different *P. larvae* genotypes have been identified, with slightly different pathological consequences at individual and colony level [5, 6]. *P. larvae* spores germinate in the midgut of young larvae, after which the bacteria breach the intestinal lining and invade the larval tissues, where they continue to proliferate and ultimately contract into billions of spores when the larval tissues are exhausted. These larval remains dry into sticky, hard scales that bees have difficulty removing and thus constitute a continuous source of new infections in successive brood cycles. This perpetual re-infection, the distribution of the spores throughout the colony by young cleaning and nursing bees, together with the hardiness and viability of the spores, are the key factors driving the lethality and within-colony epidemiology of AFB [4, 7]. The between-colony epidemiology is driven primarily by beekeepers moving contaminated material between colonies, and at a more local scale by drifting, swarming, and robbing bees [8, 9].

AFB is controlled primarily by burning symptomatic colonies, thereby destroying the long-lived spores residing in the frames and hive material, although the bees can sometimes be saved as an artificial swarm, housed on new material [4]. An alternative approach, popular in North America but illegal in Europe, is to target the germinating bacteria with antibiotics such as oxytetracycline and tylosin, thereby suppressing AFB symptoms without addressing the principal cause of the disease, i.e., the persistence of the spores. Prophylactic use of these antibiotics has inevitably led to antibiotic resistance in *P. larvae* [10, 11]. However, the strong antagonistic effects of naturally occurring lactic acid bacteria in honeybees on the infectivity and pathogenicity of *P. larvae* has identified a possible new approach to AFB control [12–15]. Beneficial bacteria belonging to the Lactobacilli and Bifidobacteria have been shown to promote honeybee health through activating the honeybee’s immune defenses [14, 16], producing antimicrobial compounds inhibiting bacterial competitors [17–19], and by outcompeting pathogenic bacteria [2, 12, 13].

The honeybee-specific Lactic Acid Bacteria (hbs-LAB) comprise a group of functionally similar bacteria that reside primarily in the honey crop [20]. Thirteen genetically distinct lactic-acid producing bacteria have been identified from the honeybee crop, of which nine are Lactobacilli and four are Bifidobacteria [20, 21]. Between then, they produce a range of metabolites, such as organic acids [22], extracellular proteins, lipopolysaccharides, and lipoteichoic acid [23] that may contribute to their inhibitory effect on *P. larvae* in microbial and infection bioassays [18, 19].

However, the honeybee colony is a complex super-organism with both individual and social immune defenses that work in tandem for managing overall colony health but lead to different consequences for infected individuals. Beneficial health effects at individual bee level do not therefore axiomatically translate into similar health benefits at colony level [24–27]. The evidence for any health benefits of manipulating the honeybee microbiome at colony level is mixed, with some studies showing largely negative effects [28], while others show more positive effects [12, 13]. We previously demonstrated the inhibitory effects of hbs-LAB on *P. larvae* infection in individual larvae [18] and that this included the secretions of antimicrobial substances that inhibit the germination of *P. larvae* spores and vegetative cell growth [29]. However, we also showed that this type of hbs-LAB formulation and administration was not effective at colony level as a curative agent for AFB-diseased colonies, since neither *P. larvae* spore levels nor AFB disease symptoms were affected throughout a whole bee season [30].

The aim of the present study was therefore to evaluate whether hbs-LAB supplements could instead function as a preventative agent, by lowering the background concentrations of *P. larvae* spores in naturally subclinically infected colonies, in apiaries with a history of AFB. A secondary aim was to characterize the natural hbs-LAB composition in colonies from apiaries with sub-clinical levels of *P. larvae*, relative to colonies in newly established “clean” apiaries, and whether supplemental administration of hbs-LAB affected the natural hbs-LAB composition in both types of colonies and apiaries. We furthermore aimed to discover a link between (cumulative) abundance and diversity of hbs-LAB and *P. larvae* abundance at colony level. The composition and amount of hbs-LAB was extrapolated from the dose-response experiments at individual larvae levels [18, 31]. Lastly, we compared the relative efficacy and reliability of two major methods for the detection and identification of hbs-LAB: Matrix-Assisted Laser Desorption/Ionization Time-of-Flight Mass Spectrometry (MALDI-TOF MS) and qPCR. This was done for a subset of eight hbs-LAB strains that could be detected by both methods.

## Methods

### Experimental Design

The experiment was performed in four honeybee apiaries located around the county Uppland with 12 colonies per apiary, all under standard commercial management. Two of the apiaries have had a history of persistent AFB (clinical outbreaks in 2012 and 2013) in all cases caused by *P. larvae* of ERIC I genotype [32]). The colonies in these apiaries were placed in quarantine management, with completely separated equipment from the remaining part of the beekeeping operation. The other two apiaries were established in 2015, with artificial swarms introduced on completely new hives and frame material and managed separately from the quarantined apiaries, with > 10 km pairwise distance between all of the apiaries, on a safe distance outside the normal flight range of honeybees. All bees and queens in the experiments were derived from the same stock and beekeeping operation. In the experiments and analyses, these two sets of apiaries and their colonies are referred to as “quarantined” and “non-quarantined,” respectively. Prior to the start of the experiment in 2016, the *P. larvae* status of all 48 colonies in the four apiaries was determined from adult bee samples collected during autumn 2015, using standard *P. larvae* spore cultivation methods [33], since spore counts from adult bee samples are the most reliable predictor of AFB disease symptoms [34]. The samples from the quarantined apiaries had between 100 and 40,000 *P. larvae* spores per honeybee while the samples from the non-quarantined apiaries were all free of *P. larvae* spores. During spring 2016, six colonies in each of the four apiaries were administered hbs-LAB supplements while the other six were given placebo supplements, in a double-blinded fashion. The supplements were administered on two occasions, in April and May 2016, coincident with the first and second sampling occasions.

### Composition of Hbs-LAB Supplements

The composition and total amount of bacteria in the hbs-LAB supplement to be used at colony level (10^6^ colony forming units (CFU)/colony) was extrapolated from previous dose-response research at individual level, in laboratory bioassays, through multiplying the optimum per-larva dose (2500 CFU/larva) [18] by the expected number of first instar larvae in a standard colony in April–May in Sweden (approximately 400). All hbs-LAB, including nine *Lactobacillus* spp. (*Lactobacillus kunkeei* Fhon2N, *Lactobacillus apinorum* Fhon13N, *Lactobacillus mellis* Hon2N*, L. mellifer* Bin4N, *L. apis* Hma11N, *Lactobacillus helsingborgensis* Bma5N, *Lactobacillus melliventris* Hma8N, *Lactobacillus kimbladii* Hma2N, *Lactobacillus kullabergensis* Biut2N) and four species/major strains of Bifidobacteria (*Bifidobacteria asteroides* Bin2N, *B. asteroides* Bin7N, *B. asteroides* Hma3N and *Bifidobacteria coryneforme* Bma6N) [20, 21], were incubated individually and anaerobically at 35 °C in Man, Rogosa & Sharpe (MRS; Oxoid, England) broth, supplemented with 2% fructose (Merck, Sollentuna, Sweden) and 0.1% l-cysteine (Sigma-Aldrich, Stockholm, Sweden) [35]. A 30-mL mixture of all 13 hbs-LAB was prepared from equal volumes of each individual bacterial strain at 10^7^ CFU/mL, and was subsequently freeze-dried in ten aliquots. Prior to feeding, the freeze-dried bacteria were resuspended in 1:10 volumes of water and incubated overnight at 35 °C, resulting in 300 μL bacterial suspension at 7.7 × 10^6^ CFU/mL for each aliquot. Each bacterial aliquot was added to 1.5 kg inverted sugar solution (Bifor®, Nordic sugar, Germany) diluted with 1 L warm water (45 °C). This mixture was incubated at 30–40 °C overnight and 10^6^ CFU (approximately 73 mL) was administered to each honeybee colony.

### Sample Management

Adult honeybees were sampled monthly from each colony between April 15 and September 15, 2016, resulting in six sampling occasions. Approximately 200 adult honeybees per sample were collected from the brood chamber in a small cardboard box. Additionally, for each colony, 20 honey crops with nectar were pulled from worker bees (see within [36]) and pooled in a 1.5-mL microcentrifuge tube containing 1 mL sterile physiological saline solution (0.9%). The honeybee samples and honey crops were stored at − 20 °C until bacterial analysis. *Paenibacillus larvae* spores are highly resistant and their viability is unaffected by freezing at − 20 °C [37].

### Cultivation of *P. larvae*

Worker honeybee samples were homogenized and cultivated on MYPGP-agar plates as described by Forsgren and Laugen [38]. Bacterial colonies were confirmed by PCR as described previously [39]. The number of *P. larvae* CFU was multiplied by the various dilution factors in the procedure to estimate the number of *P. larvae* CFU per honeybee prior to data analysis.

### Analysis of Hbs-LAB Composition in Honey Crops

The number and composition of hbs-LAB in the honey crops was assessed with two complementary methods: quantitative PCR (qPCR) and Matrix-Assisted Laser Desorption/Ionization Time-of-Flight (MALDI-TOF) Mass Spectrometry (MS). The 20 honey crops from each colony sample were homogenized in 1 mL bee physiological saline using a single-use tip. These homogenates were thereafter characterized directly by qPCR-based detection and quantification of the species/strain-specific hbs-LAB DNA in the honeybee crop, and indirectly by MALDI-TOF MS analysis of individual bacterial colonies cultured from the homogenates (see below).

### Quantitative PCR (qPCR)

DNA was extracted from 100 μL of honeycrop homogenate using the DNeasy Blood and Tissue kit (Qiagen, Germany), with minor modifications of the manufacturer’s protocol as described previously [35]. The DNA of the different hbs-LAB was quantified by qPCR using a modified version of Nadkarni et al. [40], using proprietary primers developed by ConCellae AB (Lund, Sweden) based on selective gene sequences to allow the specific detection of each of the hbs-LAB species and strains investigated here. The performance characteristics of the different assays, including detection limits, can also be obtained from ConCellae AB. The PCR reaction was performed in a total volume of 10 μL using 5 μL of Applied Biosystems SYBR green PCR master mix (Thermo Fisher Scientific, UK), 0.3 μM each of the forward and reverse primers for each hbs-LAB species and strain, and, finally, 4 μL of DNA template (10–50 ng/μL). A negative control containing water instead of DNA template was included in each run. Amplification and detection of DNA by qPCR were performed with the Quantstudio 7 Flex System (Applied Biosystems, Thermo Fisher Scientific, MA, USA) using optical-grade 384-well plates. The reaction conditions for amplification of DNA were 50 °C for 2 min, 95 °C for 2 min and 40 cycles of 95 °C for 15 s and 60 °C for 1 min. For each hbs-LAB, the absolute number of bacterial genomes, as represented by the specific gene PCRs, was calculated by the Quantstudio Software version 1.2 supplied by Applied Biosystems, using external calibration curves based on tenfold serial dilutions of DNA extracted from known amounts (in CFU) of pure culture of each hbs-LAB. The data was multiplied by the various dilution factors in the experiments to determine the estimated CFU for each hbs-LAB in each sample prior to data analysis.

### Matrix-Assisted Laser Desorption/Ionization Time-of-Flight (MALDI-TOF) Mass Spectrometry (MS)

Bacteria in the honey crop homogenates were cultured in tenfold serial dilutions on MRS agar supplemented with 2% fructose (Merck, Sollentuna, Sweden) and 0.1% l-cysteine (Sigma-Aldrich, Sweden) [31, 35]. Fifteen individual bacterial colonies from each sample were transferred to a 96-well steel target plate (Bruker Daltonics, Sweden) and covered with 1 μL 70% formic acid and 1 μL of 10 mg/mL α-cyano 4-hydroxycinnamic acid (HCCA) in a matrix solution (Sigma-Aldrich), as described previously [29]. The MS of each bacterial colony was determined by MALDI-TOF MS [41], analyzed using a Microflex instrument, the FlexControl and MALDI Biotyper 3.1 software with MBT Compass library, DB-6903 MSP (Bruker Daltonics). The resulting MS profiles of the bacterial colonies were analyzed against a combined library consisting of the MBT Compass library and an in-house reference database for the different hbs-LAB (HBS-LAB database) [21], to allow the hbs-LAB identification of each bacterial colony. A score of 2.0 or greater was needed for the positive hbs-LAB identification.

### Data Analysis

The raw data consisted of quantitative measures for the CFU per bee for *P. larvae* (determined by culturing), CFU per sample for each of the 10 hbs-LAB (determined by qPCR), and the qualitative hbs-LAB identification of individual cultured bacterial colonies (determined by MALDI-TOF MS) present in each of the 48 honeybee colonies on seven sampling occasions before, during, and after treatment with supplemental hbs-LAB or placebo. The MALDI-TOF MS data have a Bernoulli distribution (detected, not detected), whereas the *P. larvae* spore counts and qPCR have a Poisson distribution. Furthermore, the qPCR data of hbs-LAB were compressed into a Shannon diversity index capturing the strain richness (number of strains detected) and abundance of the ten hbs-LAB.

The primary objectives of the analyses were to test the effect of treatment (oral administration of hbs-LAB) and the quarantine status of the apiary on the spore counts of *P. larvae* and the presence, abundance, and diversity of hbs-LAB in the colonies. These two main objectives were pursued using the qPCR data as it offered a measure of abundance for each hbs-LAB. Although the categorization of “quarantined” and “non-quarantined” colonies was based on AFB history of the apiaries, supported by *P. larvae* spore analyses of the 2015 pre-experiment samples, the 2016 experimental samples also identified low levels of *P. larvae* spores in ten of the 135 samples from the non-quarantined colonies (Fig. S1). We have no simple explanation for these observations, other than that the *P. larvae* detection in the non-quarantined colonies may simply reflect random probabilistic detection out of a natural, low background presence. However, these findings mean that the quarantine status of a colony, or an apiary, is by itself not sufficient as an explanatory variable in the data analysis. We therefore used *P. larvae* spore counts as the primary explanatory variable for explaining hbs-LAB diversity (Table 1: Model 1; hereafter M1) or cumulative hbs-LAB abundance (Table 1: M2) in the honey crop. The main function of the quarantine/non-quarantine categories is to represent other systematic differences between the colonies in these apiaries, which consists principally of the age of the equipment (new in the non-quarantined apiaries, used in the quarantined apiaries) and secondarily of the developmental background of the colonies in 2015 (artificial swarms in the non-quarantined apiaries, established colonies in the quarantined apiaries). As recommended by Schielzeth et al. [42], the only continuous explanatory variable (“Diversity”, as represented by the Shannon index) was centered and scaled.

**Table 1:** Analysis-of-deviance tables (Type III test) from generalized linear mixed models (GLMMs) investigating the effect of hbs-LAB diversity (M1) and cumulative abundance of all hbs-LAB (M2) on the *P. larvae* spore level. Non-significant terms (italicized) were removed stepwise from the final minimal adequate model starting from the bottom row. “/” means latter random factor is nested within former random factor. The three-way interaction in M1 was removed because this interaction was mainly due to one data point having very high leverage (see text and Fig. S1)

Model	Model type	Random factor	Response variable	Explanatory variables	Χ^2^	Df	AIC	*P* value
M1	GLMM (Poisson)	Day/ Colony	*P. larvae spore level* (CFU)	Quarantine status	3.4	1	1376.3	0.06
				*Treatment*	*0.0*	*1*	*1378.3*	*0.91*
				*Diversity*	*0.2*	*1*	*1380.1*	*0.64*
				*Diversity × Quarantine status*	*0.4*	*1*	*1381.6*	*0.48*
				*Treatment × Diversity*	*0.1*	*1*	*1383.5*	*0.74*
				*Treatment × Quarantine status*	*0.0*	*1*	*1385.8*	*0.92*
				*Treatment × Diversity × Quarantine status*	*3.0*	*1*	*1414.6*	*< 0.001*
M2	GLMM (Poisson)	Day/ Colony	*P. larvae spore level* (CFU)	Quarantine status	3.4	1	1376.3	0.06
				*Treatment*	*0.0*	*1*	*1378.3*	*0.91*
				*Cumulative abundance*	*0.0*	*1*	*1380.3*	*0.93*
				*Cumulative abundance × Quarantine status*	*0.0*	*1*	*1382.3*	*0.88*
				*Treatment × Cumulative abundance*	*0.0*	*1*	*1384.3*	*0.94*
				*Treatment × Quarantine status*	*1.4*	*1*	*1385.8*	*0.22*
				*Treatment × Cumulative abundance × Quarantine status*	*24.3*	*1*	*1408.7*	*0.10*

The second series of analysis focused on the reverse effect, that of *P. larvae* spore level on hbs-LAB abundance. This was tested with a multivariate analysis of co-variance (MANCOVA) with all ten hbs-LAB as response variable (Table 2: M3). Before the backward selection, the multivariate normality of residuals was checked graphically and homogeneity of covariance matrices was checked with the Box’s M-test [43]. To meet the requirements of multivariate normality, we transformed the response variables (log (x + 1)) and as there was indication of low homogeneity in the covariance matrices we used Pillai’s trace [43]. To avoid an unnecessary complex model (e.g., indications of a temporal effect was not seen, see below) we did not include the hierarchical data structure here and used the abundances of each hbs-LAB regardless of sampling occasion and honeybee colony identity. After the multivariate model analysis, the univariate linear models for each individual hbs-LAB were extracted to check for each strain whether its individual abundance was affected by colony treatment and/or quarantine status. Furthermore, we investigated the effect of treatment on the diversity of all hbs-LAB (Table 3: M4) and the abundance of individual hbs-LAB (Table 3: M5).

**Table 2:** Multivariate analysis of co-variance (MANCOVA; Type III test) from multivariate linear model investigating the relationship between *P. larvae*, treatment and quarantine status, and the abundance of the ten hbs-LAB quantified with qPCR. Non-significant terms (italicized) were removed stepwise from the final model starting from the bottom row

Model	Model type	Response variable	Explanatory variables	Pillai’s trace	*F*	Df (num, den)	*P* value
M3	MANCOVA	Abundance (of each of the 10 hbs-LAB)	Treatment	0.02	0.74	10, 273	0.68
			Quarantine status	0.07	2.11	10, 273	0.02
			Treatment × Quarantine status	0.08	2.44	10, 273	< 0.01
			*P. larvae*	*0.03*	*0.86*	*10, 272*	*0.56*
			*P. larvae × Quarantine status*	*0.02*	*0.77*	*10, 271*	*0.64*
			*P. larvae × Treatment*	*0.01*	*0.47*	*10, 270*	*0.90*
			*P. larvae × Treatment × Quarantine status*	*0.02*	*0.71*	*10, 269*	*0.71*

**Table 3:** Analysis-of-deviance tables (Type III test) from linear mixed model (LMM) and generalized linear mixed model (GLMM) investigating the effect of hbs-LAB treatment on the hbs-LAB diversity in the honey crop (M4) and abundance of each hbs-LAB (M5). Non-significant terms (italicized) were removed stepwise from the final model starting from the bottom row. “/” means latter random factor is nested within former random factor

Model	Model Type	Random factor	Response Variable	Explanatory Variables	Χ^2^	Df	AIC	*P* value
M4	LMM	Day	Diversity (Shannon)	Quarantine status	18.8	1	970.4	< 0.001
				*Treatment*	*0.0*	*1*	*974.3*	*0.81*
				*Treatment × Quarantine status*	*0.0*	*1*	*976.8*	*0.83*
M5	GLMM (Poisson)	Day/ Colony	Individual abundance	Strain	230,430	9	1,424,487	< 0.001
				Treatment	0.3	1		0.57
				Quarantine status	6.3	1		0.01
				Strain × Treatment	52,529	9		< 0.001
				Strain × Quarantine status	17,599	9		< 0.001
				Treatment × Quarantine status	4.7	1		0.02
				Strain × Treatment × Quarantine status	41,315	9		< 0.001

The third objective was to compare the two methods (MALDI-TOF MS and qPCR) for detection of hbs-LAB. These analyses only involved data for the eight hbs-LAB that where detectable by both methods, since *L. kunkeei*, *L. apinorum*, and *L. mellifer* could not be identified by qPCR due to the lack of specific primers and *B. asteroides* Bin2N and *B. asteroides* Bin7N could not be differentiated by MALDI-TOF MS. Firstly, we compared the number of hbs-LAB detected in a sample by qPCR and MALDI-TOF MS (Table 4: M6). Secondly, the relationship of how often each hbs-LAB was detected versus not being detected in all samples from all days was analyzed (Table 4: M7).

**Table 4:** Analysis-of-deviance tables (Type III test) from generalized linear model (GLM) and generalized linear mixed model (GLMM) investigating the two methods (MALDI-TOF MS, qPCR) for detecting eight hbs-LAB that were available for both (see text). The number of different hbs-LAB detected (M6) and the relationship of detecting a hbs-LAB or not finding it (M7) were compared. Non-significant terms (italicized) were removed stepwise from the final model starting from the bottom row. “/” means latter random factor is nested within former random factor

Model	Model type	Random factor	Response variable	Explanatory variables	Χ^2^	Df	AIC	*P* value
M6	GLMM (Poisson)	Day/ Colony	Number of strains detected	Method	112.6	1	2241.8	< 0.001
				Quarantine status	23.8	1		< 0.001
				Treatment	4.4	1		0.03
				Method × Treatment	4.0	1		0.04
				*Method × Quarantine status*	*0.0*	*1*	*2243.8*	*0.95*
				*Treatment ×* Quarantine status	*0.2*	*1*	*2245.6*	*0.62*
				*Method × Treatment × Quarantine status*	*0.9*	*1*	*2246.7*	*0.33*
M7	GLM (binomial)	–	Strain detection probability (failures, successes)	Strain	206.7	7	123.9	< 0.001
				Method	44.0	1		< 0.001
				Strain × Method	140.4	7		< 0.001

All univariate models consisted of generalized models (generalized linear model, GLM) and mixed models (linear mixed model, LMM; generalized linear mixed model, GLMM). Each model started with the main factors of interest and all interactions of interest following a backwards selection until the minimal adequate model was reached. Non-significant interactions/main effects, based on the Wald chi-square tests (Type III test) and an information criterion (AIC), were stepwise removed. Models with a Binomial response (sum of successes and failures of detection for both methods) were analyzed with logit link, a Poisson response with a log link, and normal distribution with identity link function. Recommended visual inspection of residuals [44] necessitated a log-transformation in model 4 (Table 3: M4). In all mixed models, Gaussian random factors were included to model the hierarchical data structure; therefore, the repeated measure structure was accounted for. In the Binomial and Poisson models, every data pair/point received its own likelihood by nesting random factors until the observational level [45]. An autocorrelation for the sampling occasions was not included in the models because (i) preliminary analysis revealed very weak temporal correlation (e.g., M6 with covariance matrix with continuous autoregressive process: Phi = 0.2), (ii) quarantined and non-quarantined colonies should be similarly affected by time as both received either hbs-LAB treatments or placebo right after the sampling I and II, and (iii) there was no indication of a general temporal effect for any treatment-quarantine status combination (Fig. S3). The true sample size is 12 (*n* = 12 honeybee colonies) for each of the six sampling occasions (12 × 6 = 72 samples per treatment-quarantine combination). However, three non-quarantined colonies (two treated with hbs-LAB and one treated with placebo) died before the last sampling occasion, decreasing the sample size slightly.

The complete analysis was conducted using R [46]. The functions *lmer* (LMM) and *glmer* (GLMM) from the package *lme4* [47], *Anova* from the package *car* for the type III test [48], and the packages *vegan* [49], *multcomp* [50], and *lsmeans* [51] were used. The latter two packages were used to back-transform the response variables to linear scale (before calculating the arithmetic mean) and extract (Tukey method adjusted) pairwise comparisons of interest.

## Results

### The Effect of Oral Hbs-LAB Administration on Colony-Level Hbs-LAB Composition, *P. larvae* Prevalence, and their Potential Interaction

**Fig. 1:**
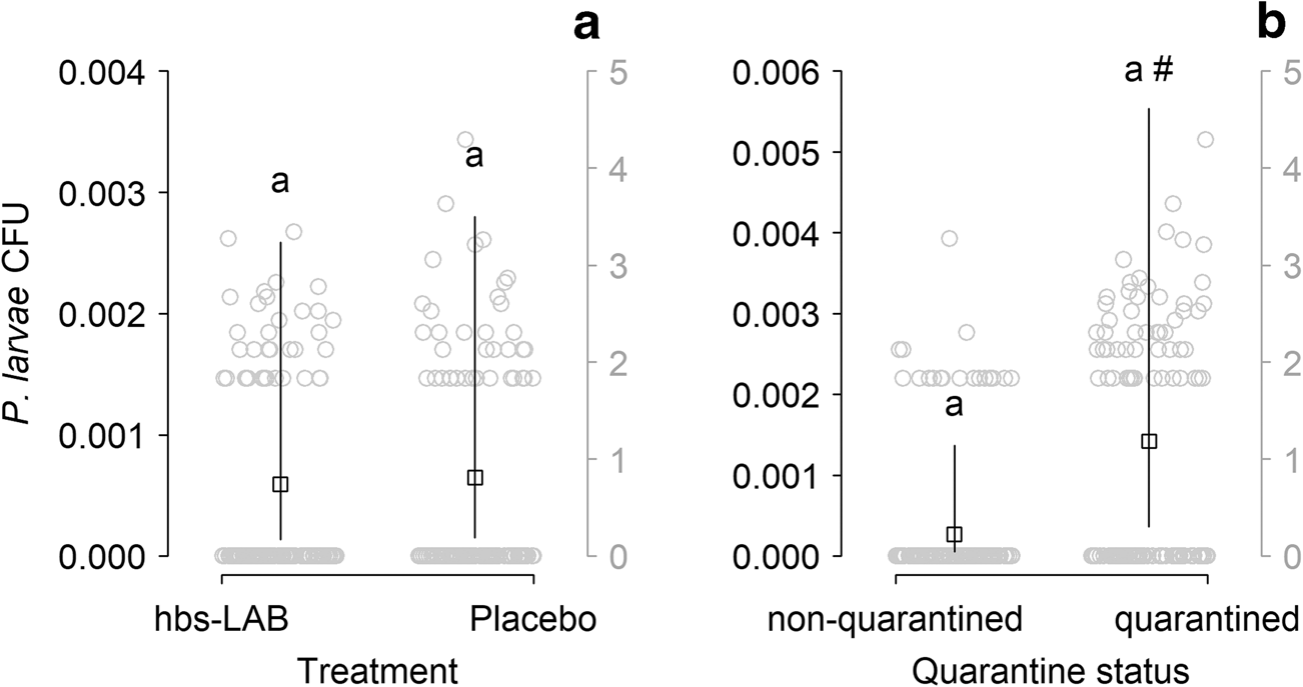
Abundance of *P. larvae*–colony forming units (CFU) found in samples from colonies regarding treatments (**A**) and/or quarantine status (**B**). The squares show the predicted marginal means with confidence limits from the statistical model. The gray dots (with a separate scale in gray on the right side) show the original data (log10 transformed after addition of one to show zero values). The hash mark indicates as strong tendency of higher *P. larvae* spore levels in quarantined colonies (*P* = 0.06)

Neither treatment with supplemental hbs-LAB (Table 1: M1 and M2, Fig. 1A), nor the hbs-LAB diversity (Table 1: M1, Fig. S1), nor the cumulative abundance of all hbs-LAB in the honey crop (Table 1: M2), nor the interaction between treatment and diversity/abundance (Table 1: M1 and M2) had an effect on the *P. larvae* spore levels. However, we found a strong tendency for higher *P. larvae* spore levels in quarantined colonies (Fig. 1B). At the beginning of model selection, we found an interaction between supplemental treatment, hbs-LAB diversity (Shannon index), and AFB quarantine status. However, this interaction was mainly due to one data point (Fig. S1) having very high leverage in the model. This interaction term was therefore removed, which considerably improved the model parsimony (AIC dropped by 28.8 points), justifying the removal. The subsequent selected model revealed no interactions.

In order to investigate the combined relationship between the various hbs-LAB and *P. larvae*, a multivariate model was run using the abundances of each individual hbs-LAB as multivariate response variables (Table 2: M3). This showed that the abundance of all hbs-LAB combined was affected by an interaction between the supplemental hbs-LAB treatment and the AFB quarantine status of the apiaries. However, investigating the univariate relationship of individual hbs-LAB abundances within the model showed no interaction effect between treatment and quarantine status, no effect of treatment and an effect of quarantine status for all individual hbs-LAB abundances except for *L. kimbladii* Hma2N and *B. asteroides* Bin2N (Table S1). Furthermore, no relationship between the abundance of any individual hbs-LAB and *P. larvae* spore levels was noticed.

### The Effect of Oral Hbs-LAB Administration on Hbs-LAB Diversity and Individual Abundance in the Honey Crop

**Fig. 2:**
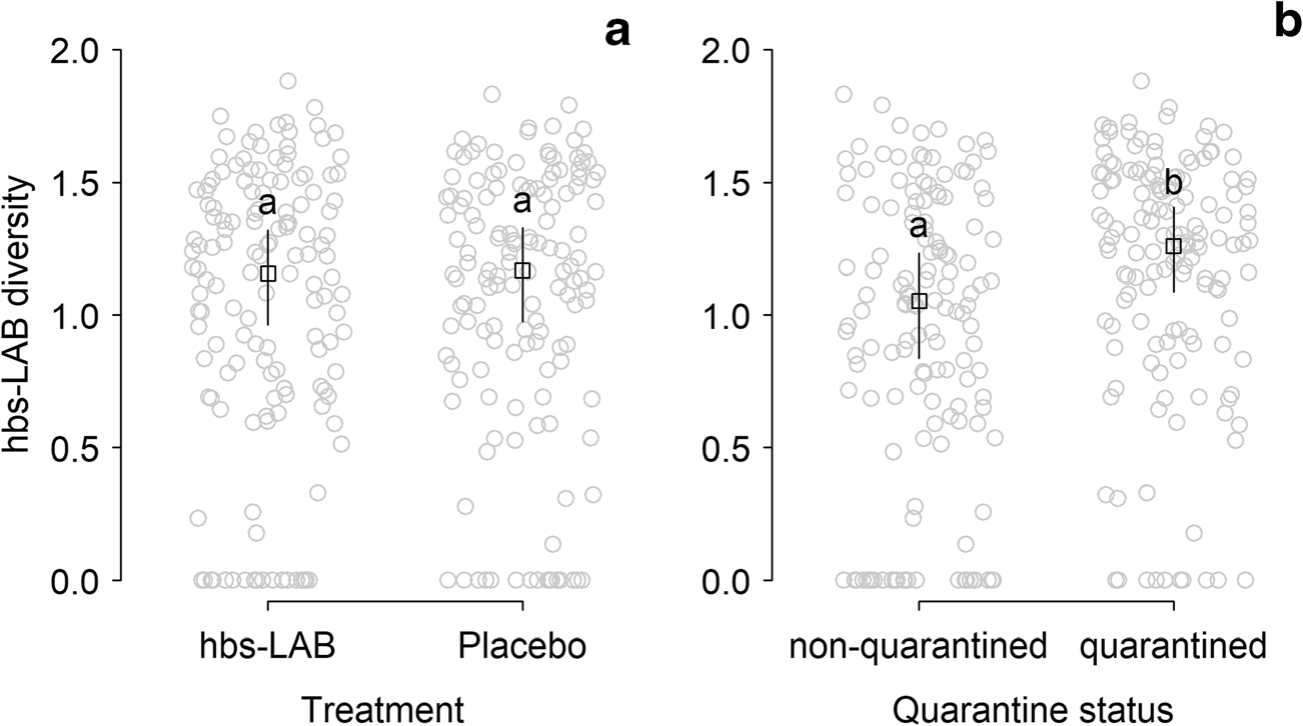
Hbs-LAB diversity (Shannon index) found in samples from colonies regarding treatments (**A**) or quarantine status (**B**). Different lower-case letters indicate significant differences between predicted marginal means with confidence limits while gray dots represent the original diversity of all sampling occasions of all colonies

The diversity of hbs-LAB in the honey crop was unaffected by supplemental treatment in either the quarantined and non-quarantined apiaries, but we found significantly higher diversity (around 19%) in quarantined colonies (Table 3: M4; Fig. 2; see also Fig. S2 for variability of diversity over time with regard to treatment and quarantine status).

**Fig. 3:**
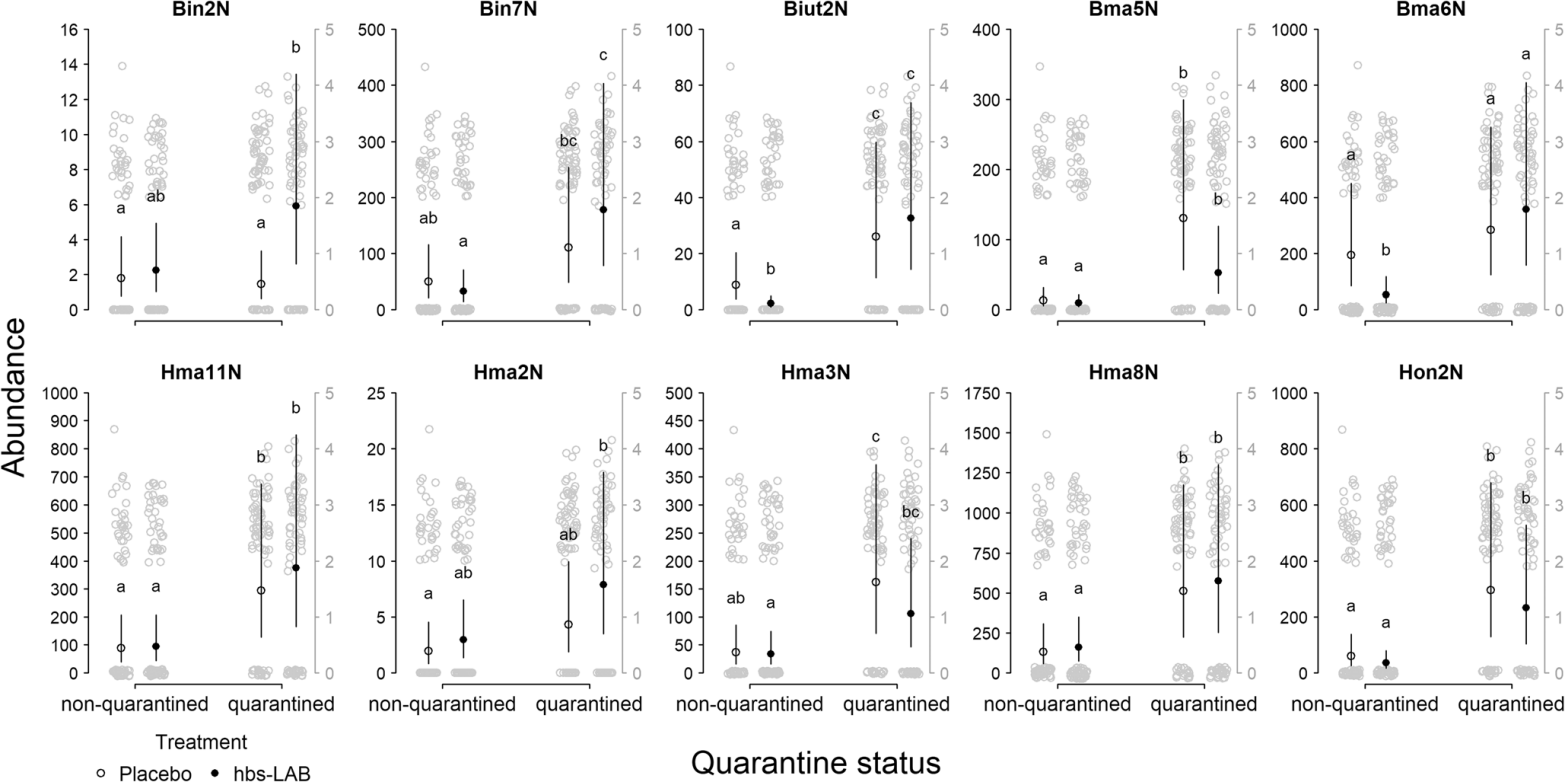
Abundance of each hbs-LAB found in a sample with qPCR. The black dots show the predicted marginal means with confidence limits from the statistical model. The gray dots (with a separate scale in gray on the right side) show the original data (log10 transformed after addition of one to show zero values). Different letters indicate significant differences between treatments-quarantine status combinations within each hbs-LAB

Only weak support was observed for an effect of supplementation of hbs-LAB on changes in the abundance of individual hbs-LAB (Fig. 3). Since the model showed a significant three-way interaction between strain, treatment, and quarantine status (Table 3: M5), we extracted the comparisons of interest (treatment-quarantine status combinations within each hbs-LAB) from all 780 comparisons. Only in one case (quarantined: *B. asteroides* Bin2N) we found a higher abundance in the hbs-LAB treatment. Further illustrating the absence of a supplementation-abundance relationship is the fact that in two cases (non-quarantined: *L. kullabergensis* Biut2N, *B. coryneforme* Bma6N), we observed a higher abundance in the placebo treatment. However, we found strong support for higher hbs-LAB abundance if colonies had a history of infection (quarantined colonies). For example, of the 20 comparisons (10 strains, 2 treatments), 14 showed significantly higher abundance if the colony was quarantined.

### Comparison of MALDI-TOF MS and qPCR for Detecting Individual Hbs-LAB

**Fig. 4:**
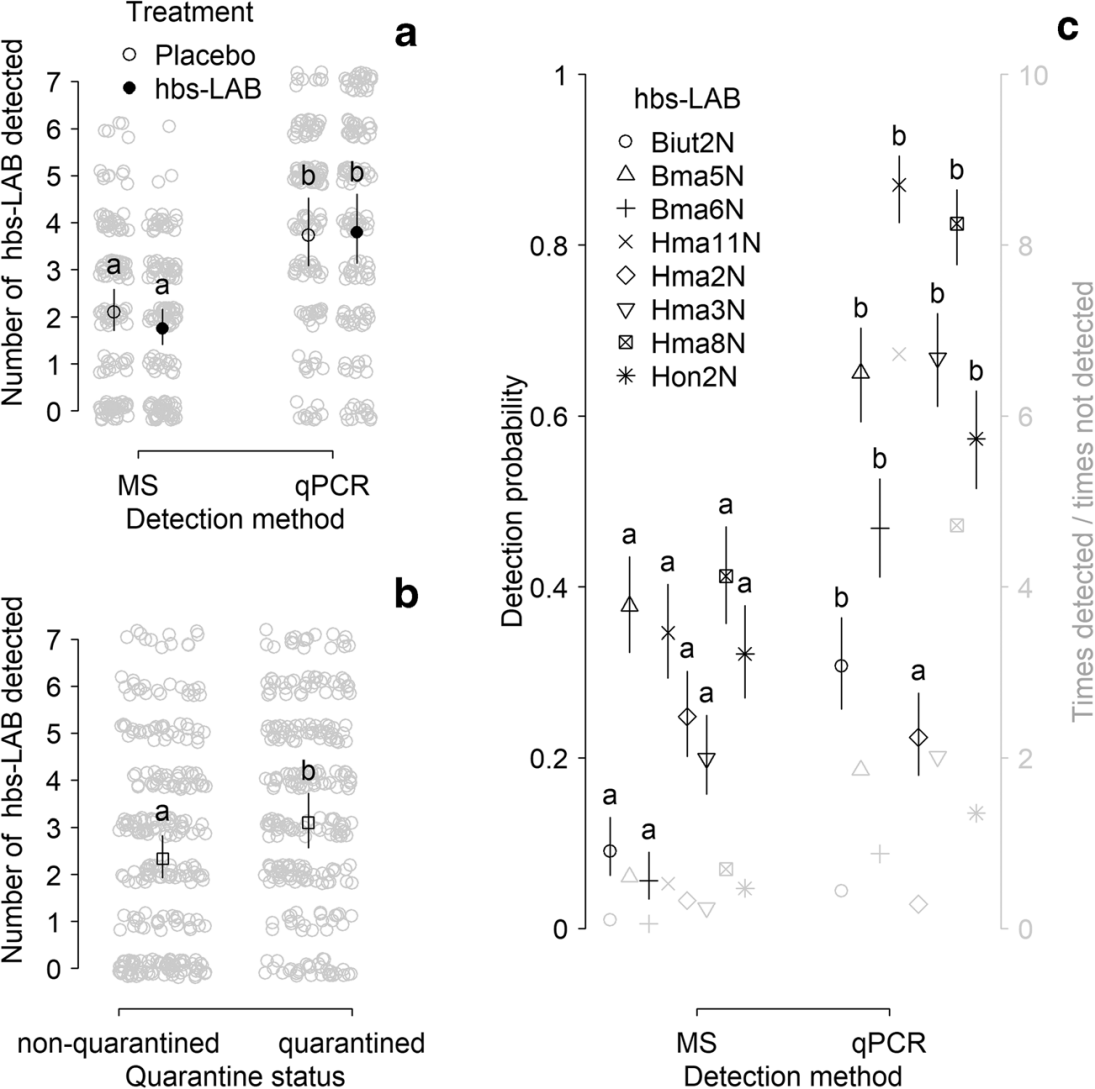
The number of different hbs-LAB found in a sample was greater using qPCR technique (**A**) and in the quarantined honeybee colonies (**B**). Individual hbs-LAB was significantly better detected using qPCR in comparison with MALDI-TOF MS, except Hma2N that was detected similarly with both methods (**C**). Different lower-case letters indicate significant differences between predicted marginal means with confidence limits form the respective models. In **A**, all four combinations are compared regardless of quarantine status; in **B**, quarantine status is compared regardless of treatment-methods, and in **C**, different methods are compared for each hbs-LAB respectively. In **A** and **B**, the gray circles show the original data (all colonies at all sampling occasions) while in **C**, the gray symbols show the original ratio of detection (times detected divided by times not detected)

Based on the number of detecting or not detecting different hbs-LAB, we could compare the two most common techniques, qPCR and MALDI-TOF MS for detection and identification of these bacteria using eight strains that were detectable with both methods. The number of hbs-LAB detected in a sample was affected by the method-treatment interaction while all other interactions between the method, the treatment, and the quarantine status were not significant (Table 4: M6). However, pairwise comparisons of the mean values did not show differences between treatments, and regardless of quarantine status more hbs-LAB could be detected using qPCR (Fig. 4A). Moreover, we detected generally more hbs-LAB in the quarantined colonies independent of the method (Fig. 4B).

Investigating how often each particular hbs-LAB was detected or not in all samples showed that seven out of eight hbs-LAB had higher detection probability using qPCR compared to MALDI-TOF MS. The model resulted in an interaction between strains and method, which can be explained by the different detection probabilities with the two methods (Table 4: M7; Fig. 4C; see Table S2 for comparisons among all method-strains combinations). The only hbs-LAB that was not more efficiently detected by qPCR, was *L. kimbladii* (Hma2N); in this case both techniques were equally good. The greatest difference was found for *L. apis* (Hma11N) that was detected up to approx. sevenfold more often than not being detected (249 times detected /37 times not detected; Fig. 4C), which represented an approx. 12-fold better detection with qPCR compared to MALDI-TOF MS ((249/37)/(99/187)).

## Discussion

Bacteria in the genera *Lactobacillus* and *Bifidobacterium* produce a range of antimicrobial metabolites, such as organic acids, antimicrobial peptides, and bacteriocins [17, 52–54]. It is therefore not surprising that their potential for supporting honeybee health, by limiting the virulence and spread of pathogens, has received much attention. However, only very few relevant field studies have been conducted on the effects of feed supplements containing beneficial bacteria on honeybee colony health and performance [12, 17, 28, 30].

In our experiments we showed that feed supplements containing hbs-LAB had no effect on hbs-LAB composition and abundance in the honey crop. We further verified our previous finding that *P. larvae* spore levels in the colonies are not altered by administration of hbs-LAB [30]. However, we did observe that hbs-LAB diversity (+ 19% Shannon index, higher number of strains) was much higher in quarantined colonies (i.e.*,* those with a history of an AFB outbreak) than in non-quarantined colonies (i.e.*,* those without a history of AFB) although hbs-LAB diversity was otherwise unrelated to *P. larvae* spore levels. This can have several possible explanations. Since the observations are coincidental with quarantine status, they could be related to any systematic difference between the colonies in the two sets of apiaries. This includes not only AFB history and *P. larvae* spore levels but also differences in the age of the bee equipment, colony establishment in 2015, geography and microclimate, different foraging resources, etc. Excluding all these alternatives, will require dedicated experimentation. However, qualitative and quantitative changes in the honeybee symbiotic microbiome due to pathogen pressure has been reported previously, in relation to *P. larvae* infection [55], *Melissococcus pluton* (the infective agent of European foulbrood) [56] and *Varroa destructor* infestation [57, 58]. It has been shown that *B. asteroides* abundance decreased in symptomatic *P. larvae*–infected honeybee colonies [55], while the proportions of *L. kunkeei*, *B. coryneforme*, and *L. mellis* were higher in symptomatic *M.* –*pluton* infected honeybees [56]. Furthermore, the abundance of *L. mellis*, *L. kunkeei*, *L*. *kullabergensis*, *L. helsingborgensis*, and *L. kimbladii* increased with increasing levels of varroosis [57]. These results suggest that the hbs-LAB and composition in adult honeybees may be a factor in brood diseases, even if the adult honeybees themselves are not affected [59], either through altering the ability of adult bees to engage in social immune responses (hygienic behavior) or through changes in the bacterial composition driven by bacterial infections in the brood; acquired and transmitted by adult bees through hygienic behavior and social interactions. It is only in the larvae that *P. larvae* and hbs-LAB can directly influence each other. The initial composition of the hbs-LAB that newly hatched larvae are exposed to would reflect its composition in the adults. Any subsequent change in adult bee hbs-LAB composition due to *P. larvae* infection in the larvae would have to be filtered back to the adult bee population through hygienic behavior and other interactions between adults and larvae, in order to affect the hbs-LAB composition at the colony level. There are a number of ways by which this could theoretically happen, but these are indirect and long-term, possibly involving a combination of innate larval immunity, adult hygienic behavior and colony-level population turnover. Furthermore, hbs-LAB composition changes during the season, depending on the flowers visited and the honeybees’ health status [20]. These bacteria produce different metabolites such as organic acids [22], peptides, and proteins that are species or even strain-specific [23]. For example, *L. kunkeei* is the most dominant hbs-LAB isolated from honey crops, honey, pollen, and beebread during spring and summer [20, 31, 56, 60], but is practically absent during the winter. It has been noticed that *L. kunkeei* produces a more diverse range of unknown proteins with putative antimicrobial activities than the other hbs-LAB [23]. Furthermore, we previously showed that hbs-LAB produce antimicrobial substances, which can inhibit the growth of *P. larvae* spores and vegetative cells at the individual level [29]. We can therefore not exclude the possibility that administration of one highly potential hbs-LAB may have a practically relevant effect in form of lowering *P. larvae* spore levels. It can, however, be debated whether supplemental administration of hbs-LAB can ever be effective for managing pathogens at the colony level given what we have shown here and previously [30], i.e., that these effects do not occur at the colony level. Hence, any promising results of the effects of hbs-LAB on pathogens at the individual level in controlled laboratory experiments [18] do not necessarily translate directly into an effective treatment for honeybee colonies.

The absence of a colony-wide effect could be explained by multiple reasons. In previous studies, an inverse relationship between the individual- and colony-level virulence for the two principal genotypes of *P. larvae* has been observed. The more virulent genotype at the individual level (ERIC-II) kills the larvae before they are capped, such that they can be easily detected and removed by social behaviors resulting in a reduced virulence at the colony level. The reverse is true for the ERIC-I genotype, whose reduced virulence at an individual level allows infected larvae to be capped, escaping early detection and removal, such that the infection finishes with spore production in hard sticky scales and thus a higher virulence at colony level [61]. By similar reasoning, those larvae with strong individual immune defenses that delay infection may therefore compromise the efficacy of the social defenses relative to larvae with weaker individual immunity [25]. Moreover, honeybee colonies have multiple homeostatic mechanisms for managing colony health and performance, of which, hbs-LAB are a small component. The close hbs-LAB-host symbiotic relationship is naturally recalcitrant to any outside efforts at manipulating either the abundance or the composition of the hbs-LAB. The contrasting results between individual and colony level experimentation highlight the strength of the honeybee homeostatic mechanisms in neutralizing the effects of any attempt to manipulate the natural conditions of the nest [62, 63]. Other studies have also shown that using beneficial microbes for treatment does not have the expected positive effects [17, 28, 64]. For example, Maggi et al. showed that supplying colonies with organic acids produced by the bacterium *Lactobacillus johnsonii* CRL1647 did not change the disease dynamics of *Nosema* spp. at the colony level [17]. Pretreatment with *Snodgrassella alvi* made honeybees more susceptible to the protozoan *Lotmaria passim*, suggesting that the probiotic therapy has complicated consequences for parasite susceptibility, microbiota homeostasis, host developmental, and detoxification response pathways [64]. Ptaszyńska et al. revealed that supplementing honeybee diets solely with the commercial probiotic, *Lactobacillus rhamnosus* with/without the prebiotic inulin, increased mortality levels in *Nosema ceranae*–infected honeybees [28]. It was recently shown that hbs-LAB may have short-term negative effects on the brood [30] although no negative effects of oral hbs-LAB administration like increased susceptibility (*P. larvae* spore counts higher in the hbs-LAB treatment) or elevated mortality among the quarantined colonies could be observed in this study. Whether more frequent hbs-LAB administration or higher dosages would have made much difference need further studies [27].

The comparison of the two different methods for detecting, identifying, and quantifying hbs-LAB (MALDI-TOF MS and qPCR) showed that qPCR could detect a wider range of hbs-LAB, with higher specificity and sensitivity than MALDI-TOF MS, although in specific cases, MALDI-TOF could differentiate hbs-LAB *(*e.g., *L. kunkeei*, *L. apinorum*, and *L. mellifer*) that qPCR could not. It should be noted that one of the drawbacks of qPCR assays of bacterial DNA is that it does not distinguish between dead and alive cells, meaning that the bacterial composition measured by qPCR may not completely reflect the living community. Using bacterial mRNA as an (RT)-qPCR template allows for only alive cells to be assessed, but is less reliable for quantifying the number of CFU, due to the confounding effect of differential RNA expression levels on cellular quantification. Although MALDI-TOF MS itself is relatively simple, quick, and cheap to perform, it requires that the bacteria can be cultured on laboratory media prior to analysis, which may differ for different types of bacteria. Consequently, also analysis by MALDI-TOF MS may not completely reflect the living community. Moreover, MALDI-TOF MS still requires supplementary methods, such as 16S rRNA sequencing, to differentiate closely related species or strains [65]. In this study, two closely related strains of *B. asteroides* (Bin2N and Bin7N) that could be differentiated by qPCR were indistinguishable using MALDI-TOF MS. Since all methods have their biases, a fully comprehensive analysis of (hbs-LAB) microbial communities should therefore use multiple complementary methodologies [2].

In conclusion, we showed that the hbs-LAB composition of honeybees is more diverse in honeybee colonies with elevated *P. larvae* spore levels, but we found no support that the hbs-LAB composition in bees can be altered by this type or dose of hbs-LAB supplements. This work does not refute the beneficial nature of hbs-LAB found in the honey stomachs of honeybees, as abundantly shown previously [18, 19, 22, 31]. However, we did not find any effect of similar supplementary hbs-LAB administration on the *P. larvae* spore levels at colony level, highlighting that data and conclusions derived from laboratory experiments on individual bees may not be applicable for the colony [2, 27].

## Electronic supplementary material

ESM 1 (DOCX 129 kb)

## Data Availability

The datasets generated during and/or analyzed during the current study are available from the corresponding author on reasonable request.
