# Supplementary material for: Feeding Honeybee Colonies with Honeybee-Specific Lactic Acid Bacteria (Hbs-LAB) Does Not Affect Colony-Level Hbs-LAB Composition or Paenibacillus larvae Spore Levels, Although American Foulbrood Affected Colonies Harbor a More Diverse Hbs-LAB Community
Source: Microb Ecol. 2019 Sep 10;79(3):743–55. doi: 10.1007/s00248-019-01434-3 (PMC7176604; doi:10.1007/s00248-019-01434-3)
Supplement: Supplementary file 1 — (DOCX 129 kb) [file 248_2019_1434_MOESM1_ESM.docx]

Electronic Supplementary Material for the article:

**Feeding honeybee colonies with honeybee specific lactic acid bacteria (hbs-LAB) does not affect colony-level hbs-LAB composition or *Paenibacillus larvae* spore levels, although American foulbrood affected colonies harbor a more diverse hbs-LAB community**

**Sepideh Lamei^1,2^, Jörg G. Stephan^1,3*^, Bo Nilson^4,5,^, Sander Sieuwerts^6^, Kristian Riesbeck^2^, Joachim R. de Miranda^1^ and Eva Forsgren^1^**

^1^ Department of Ecology, Swedish University of Agricultural Sciences, Uppsala, Sweden

^2^ Department of Translational Medicine, Lund University, Malmö, Sweden

^3^ Swedish Species Information Centre, Swedish University of Agricultural Sciences, Uppsala, Sweden

^4^ Clinical Microbiology, Labmedicin, Region Skåne, Lund, Sweden

^5^ Department of Laboratory Medicine Lund, Lund University, Lund, Sweden

^6^ Arla Innovation Center, Aarhus, Denmark

*For correspondence. E-mail jorg.stephan@slu.se


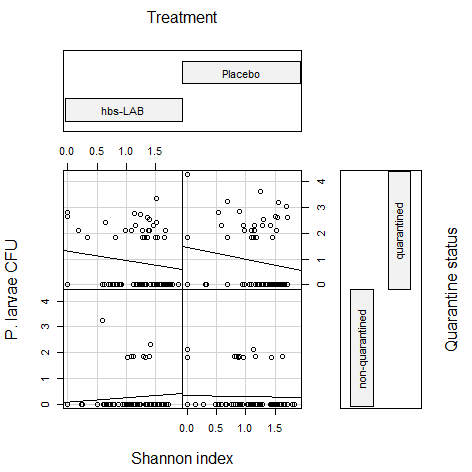


**Figure S1** Abundance of *P. larvae* (log10 transformed after addition of one) in relation to hbs-LAB diversity for the treatment and quarantine status. Lines indicate linear predictions from raw data.**Table S1** P-values from univariate test from the MANCOVA (M3).

| Strain | Treatment | Quarantine status | Treatment × Quarantine status |
| --- | --- | --- | --- |
| Bin4 | 0.46 | 0.02 | 0.18 |
| Hon2 | 0.36 | 0.00 | 0.25 |
| Hma11 | 0.69 | 0.01 | 0.55 |
| Bma5 | 0.96 | 0.00 | 0.67 |
| Hma2 | 0.88 | 0.34 | 0.32 |
| Biut2 | 0.77 | 0.00 | 0.20 |
| Bin2 | 0.71 | 0.79 | 0.38 |
| Bin7 | 0.46 | 0.00 | 0.24 |
| Hma3 | 0.73 | 0.03 | 0.75 |
| Bma6 | 0.42 | 0.02 | 0.21 |


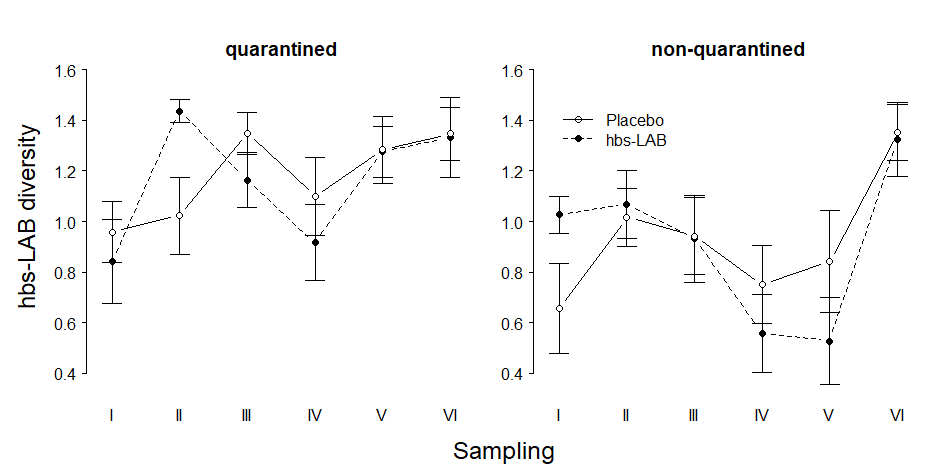
 **Figure S2** Raw data mean (± 1 SE) of hbs-LAB diversity (Shannon index) over time with regard to the quarantine status and hbs-LAB treatment. Hbs-LAB or placebo treatment was applied immediately after sampling occasions I and II.**Table S2** Letter display for pairwise comparisons between hbs-LAB detected with MALDI-TOF MS or qPCR. Among the comparisons for a particular hbs-LAB all comparisons indicate significant differences except *L.* *kimbladii* Hma2.

| Strain | MS | qPCR |
| --- | --- | --- |
| Biut2 | eh | i |
| Bma5 | b | def |
| Bma6 | cd | i |
| Hma11 | a | deg |
| Hma2 | gh | fgh |
| Hma3 | b | h |
| Hma8 | a | de |
| Hon2 | bc | eh |


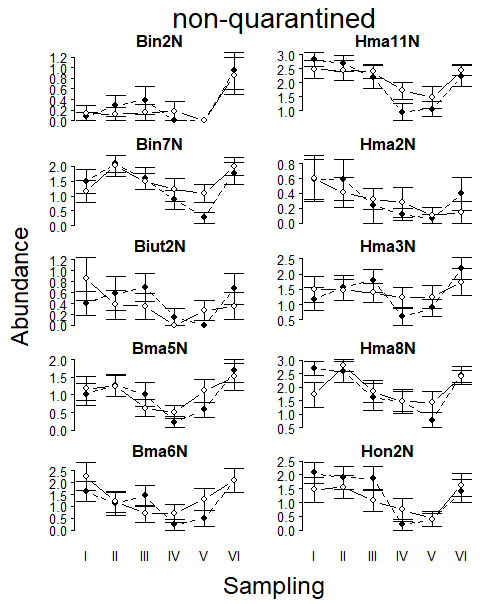

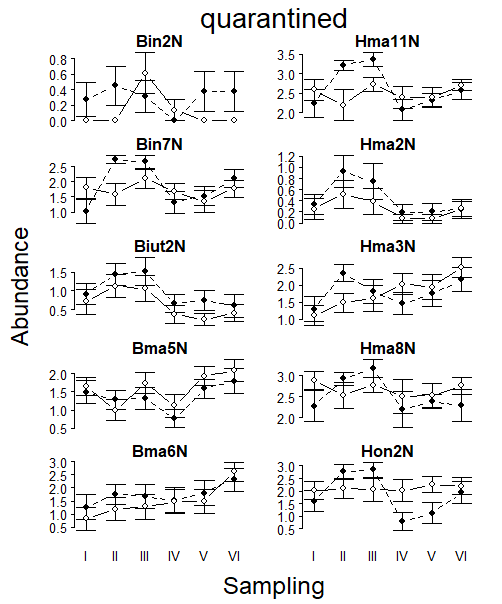


**Figure S3** Raw data means (log10 transformed after addition of one; ± 1 SE) of abundance of each hbs-LAB detected with qPCR with respect to sampling occasion, quarantine status and hbs-LAB treatment which was applied right after sampling I and II, respectively.
